# Implication of lipid metabolism disturbance and Alzheimer’s disease: focus on the lipoprotein lipase plays an important role in learning and memory function

**DOI:** 10.1186/1750-1326-7-S1-O9

**Published:** 2012-02-07

**Authors:** Dehua Chui, Ting Zhou, Liang Zhou, Huan Yang, Xinying Liu, Tingting Liu, Jia Yu, Yi Liu, Xue Fei Wu, Hui Zhang, Dongsheng Fan

**Affiliations:** 1 Peking University Neuroscience Research Institute, Dep. of Neurology, Peking University Third Hospital, Beijing 100191, China

## Background

Along with aging, lipid metabolism disturbance occurs. Lipoprotein lipase (LPL) is also expressed in the brain with highest levels found in the pyramidal cells of the hippocampus, suggesting a possible role for LPL in the regulation of cognitive function. However, very little is currently known about the specific role of LPL in the brain.

## Method

LPL deficient mice and littermate control C57BL/6J mice were bred in the animal facility of Peking University Health Science Center. The histochemistry and western blotting was performed as our previous description.

## Results

In this study, we found that LPL-deficient mice exhibited increased latency to escape platform and increased mistake frequency. Decreased latency to platform in the step-down passive avoidance test was observed, consistent with impaired learning and memory in these mice. Transmission electron microscopy revealed a significant decrease in the number of pre-synaptic vesicles in the hippocampus of LPL-deficient mice. The levels of the pre-synaptic marker synaptophysin were also reduced in the hippocampus while post-synaptic marker PSD-95 levels remained unchanged in LPL-deficient mice. LPL deficiency reduced the frequency of miniature excitatory postsynaptic currents (mEPSCs) and readily releasable pool (RRP) size. Then we demonstrated that these defects, which resulted from slower clathrin-mediated endocytosis and synaptic vesicle recycling, led to presynaptic dysfunction and synaptic plasticity impairment. Moreover, lipid assay revealed a lack of docosahexaenoic acid (DHA) and arachidonic acid (AA) in LPL deficient neurons. Meanwhile, exogenous DHA and AA partially rescued the defect of synaptic vesicle recycling in LPL deficient neurons.

## Conclusion

This finding reveals a novel role of LPL in synaptic plasticity and contributes to a better understanding of the LPL function in the brain, where altered LPL levels are related to learning and memory impairment.

